# "One-way" clinical extubation: an alternative to tracheostomy or palliative extubation in patients receiving inappropriate mechanical ventilation?

**DOI:** 10.62675/2965-2774.20260248

**Published:** 2026-03-03

**Authors:** Sabrina Corrêa da Costa Ribeiro, Ana Laura Jardim Tavares, Fernanda Gomes Lopes, Fernanda Palmas Fernandes Greco, Daniel Neves Forte

**Affiliations:** 1 Universidade Federal do Ceará Fortaleza CE Brazil Universidade Federal do Ceará - Fortaleza (CE), Brazil.; 2 Universidade de São Paulo Hospital das Clínicas Faculdade de Medicina São Paulo SP Brazil Hospital das Clínicas, Faculdade de Medicina, Universidade de São Paulo - São Paulo (SP), Brazil.; 3 Universidade de Fortaleza Fortaleza CE Brazil Universidade de Fortaleza - Fortaleza (CE), Brazil.

An 82-year-old man with mild dementia was admitted with acute respiratory failure due to pneumonia. He was drowsy, hypotensive, and hypoxemic, requiring intubation and vasopressors. After 10 days, despite some clinical improvement, he remained intubated and neurologically impaired, with persistent hypoactive *delirium*.

The medical team faced a dilemma: his high risk of extubation failure clashed with his family's emphasis on his value for independence. His family worried that prolonged dependence would contradict his preferences. The team debated how long to continue mechanical ventilation, considering the uncertain functional recovery. Should they attempt extubation despite a high risk of failure and potential reintubation or tracheostomy? What level of intervention would balance clinical judgment and patient values if his condition worsened? In patients without a terminal disease, should palliative extubation be considered?

## INTRODUCTION

In Brazil, withdrawal of life-sustaining therapy (LST) is regulated by resolution 1806/2006 of the *Conselho Federal de Medicina*,^(1)^ which considers it legal and ethical in incurable illnesses when consistent with the patient's or surrogate's wishes. However, this might not fully apply to patients with acute conditions worsened by chronic illnesses like advanced frailty or progressive, non-terminal diseases.

Mechanical ventilation is often central to these decisions. Even after a successful spontaneous breathing trial, 10 - 20% of patients fail extubation^(2)^ and require reintubation or tracheostomy. Risks are higher in the elderly, chronically ill, frail, or those on prolonged mechanical ventilation,^(2)^ with reintubation carrying mortality rates up to 50%.^(3)^ These data call for careful reflection on intensive care unit (ICU) goals and realistic long-term outcomes.

After critical illness, some patients recover, others die, and a third group stabilizes but remains LST-dependent,^(4)^ often with decreased consciousness and functional impairment, incompatible with what they would consider acceptable quality of life. In patients at high risk of extubation failure, the ethical dilemma arises: continue life support despite outcomes the patient would have declined, or consider palliative extubation, even when clinical criteria for extubation are met and despite its limited discussion in non-terminal cases?

### A Third Option: One-Way Clinical Extubation

One-way clinical extubation can be defined as the withdrawal of mechanical ventilation in a patient who:

1. Has an above-average risk of extubation failure and in-hospital mortality.
2. Meets conventional respiratory but not neurological criteria for extubation.
3. Is deemed reasonably feasible for extubation by the care team; and
4. Has a previously established advance care plan (ACP) including do not intubate (DNI)/do not resuscitate (DNR) orders.

### The Need for a Third Definition

In conventional extubation, neurological status is central. One-way extubation is less restrictive: impaired consciousness raises the risk of failure but does not preclude extubation if respiratory criteria are met. Crucially, one-way extubation must always follow a goals-of-care discussion, accompanied by a DNI/DNR order in place.

In palliative extubation, ventilation is withdrawn to honor patient wishes, prioritize comfort, and allow natural death, with mortality near 77% and median survival of 8,9 hours.^(5)^ In contrast, one-way extubation is a novel practice with no published outcome data. However, in our experience, more patients survive ICU discharge compared to the high early mortality seen with palliative extubation.

We consider one-way clinical extubation a form of proportional care, rather than withdrawal of life support, since weaning follows standard protocols and extubation criteria are met. If failure occurs, however, reintubation would be inconsistent with meaningful recovery and deemed potentially inappropriate under CREMESP Resolution 355/2022.^(1)^

Differences between conventional, one-way and palliative extubation are summarized in table 1.

**Table 1:** Differences between standard clinical extubation, one-way extubation, and palliative extubation

	Clinical extubation	One-way extubation	Palliative extubation
Patient eligibility criteria	Extubation failure risk is deemed to be average Patient fulfills ventilatory and neurological extubation criteria	Risk of extubation failure is deemed to be higher than average Patient fulfills ventilatory criteria (but not necessarily neurological ones) for extubation	Patient has a terminal illness and ventilatory support is considered inappropriate
Primary clinical goal	Withdraw mechanical ventilation when a patient has recovered from acute illness and no longer needs ventilatory support	Withdraw mechanical ventilation in a patient who has improved enough to fulfill ventilatory criteria but has a higher-than-average risk of failure	Interrupt mechanical ventilation when it does not fulfill its goal or is incompatible with patient values
Anticipated outcomes	Complete or partial recovery	Reasonable possibility of survival	Most likely outcome is death within hours or days
Procedural steps	Extubate according to usual weaning protocols	Identify the eligible patient and align the goals of care in a family meeting. Reduce ventilatory parameters. Extubate the patient in the best possible condition, tolerating decreased consciousness if reversible causes have been addressed	Identify the eligible patient, align goals of care in a family meeting, and define that continued ventilatory support is technically inappropriate and/or incompatible with patient values. Extubate as soon as possible, regardless of clinical stability, with patient comfort as the primary goal
Role of advanced care planning	Plan for future care	Define the following steps should respiratory failure ensue	Define future steps if the patient survives extubation
Post extubation care planning	Rehabilitation Reintubation in case of failure	Rehabilitation in the best-case scenario Comfort care in case of failure	Comfort care

### Ethical and Multidisciplinary Aspects

For patients with advanced non-terminal illnesses, when invasive support is deemed inappropriate by clinicians and surrogates, one-way extubation provides a middle ground between LST and palliative extubation. It seeks recovery while honoring patient values and prepares all involved for the possibility of death if improvement does not occur. Rooted in prudence - understood since Aristotle as sound judgment amid risk - it balances hope with dignity in every outcome. It also reflects Kant's view of autonomy, in which the end of life is a moment to recognize and honor the principles that guided one's life.^(6)^

The multidisciplinary team plays a key role. Decisions should be collaborative, guided by patient goals, and communicated with clarity and compassion.^(7)^ Although these moments often bring fear, sadness, or uncertainty to professionals, they may also generate hope and relief when choices reflect patient-centered values. Open dialogue reduces moral distress, builds confidence, and upholds dignity by ensuring every team member feels heard.

### Practical Aspects

A patient-centered, stepwise approach to one-way extubation involves optimizing recovery, assessing readiness, and minimizing failure risk through infection control, fluid and electrolyte management, *delirium* and pain treatment, and structured weaning protocols. Once optimal conditions are reached, extubation is performed.

A plan must be defined in advance. In standard practice, this usually includes reintubation if the initial attempt fails. In one-way extubation, however, the focus shifts to comfort - prioritizing symptom control and supportive care, while helping the team and family understand that failure to maintain breathing may reflect the natural course of dying.

### Limitations and Potential Risks

The proposal for this new definition has limitations. There are no empirical data on potential benefits, and one-way extubation is not widely adopted in ICU practice. Still, a formal definition could guide implementation and generate standardized evidence on its effectiveness.

Risks must also be acknowledged. Physicians often offer pessimistic prognoses,^(8)^ which may bias patient selection. There is also the danger of using one-way extubation as a "softer" alternative when palliative extubation would be more appropriate, prolonging the dying process and increasing costs and suffering.

Besides, current experiences come from teams with dual expertise in intensive and palliative care. Widespread use by less experienced teams could lead to conceptual confusion and miscommunication. For safety reasons, this approach should be considered only within a structured, ethically guided, multidisciplinary framework.

### The Role of Patient Autonomy and Advance Care Planning

Unlike in the US, Canada, Australia, and much of Europe, palliative extubation remains uncommon in Brazil.^(9)^ Conversations about death are infrequent, advance care planning is rare, and decisions often occur in emergencies, when patients cannot participate. Families, fearing abandonment, frequently consent to intubation for severely frail patients without prior goals-of-care discussions.

In the absence of expressed patient values, clinicians and families reconstruct them indirectly - drawing on biography, past choices, preferences, and experiences of suffering. Many families highlight loss of mobility or daily autonomy as a significant source of suffering. For these patients, one-way extubation may resonate: it allows families to preserve hope without committing to prolonged life support that conflicts with patient values, while recognizing extubation failure as a sign that the body may no longer recover.

## CONCLUSION

Defining one-way extubation clarifies an alternative pathway and enables future research comparing functional and mortality outcomes for patients at high risk of extubation failure. It helps distinguish the impact of one-way extubation from both palliative extubation and continued invasive support without structured goals-of-care discussions.

This definition is not definitive; instead, it aims to stimulate debate, reflection, and the development of more precise clinical guidance for patients with uncertain trajectories.

## Data Availability

The contents are already available.
